# An unusual potassium conductance protects *Caenorhabditis elegans* pharyngeal muscle rhythms against environmental noise

**DOI:** 10.1073/pnas.2422709122

**Published:** 2025-04-03

**Authors:** Max Kenngott, Piali Sengupta, Shawn Lockery, Eve Marder

**Affiliations:** ^a^Volen Center for Complex Systems, Brandeis University, Waltham, MA 02453; ^b^Department of Physics, Brandeis University, Waltham, MA 02453; ^c^Department of Biology, Brandeis University, Waltham, MA 02453; ^d^Institute of Neuroscience, University of Oregon, Eugene, OR 97403

**Keywords:** rapid K channel inactivation, plateau properties, synaptic noise

## Abstract

The nematode *Caenorhabditis elegans* feeds by rhythmic contraction and relaxation of a neuromuscular organ called the pharynx, which draws in and filters water and bacterial food. This behavior is driven by myogenic plateau potentials, long-lasting depolarizations of the pharyngeal muscle, which are timed by neuronal input from a dedicated pharyngeal nervous system. While the timing of these plateaus’ initiation has received significant attention, their mechanisms of termination remain incompletely understood. In particular, it is unclear how plateaus resist early termination by hyperpolarizing current noise. Here, we present a computational model of pharyngeal plateaus against a noisy background. We propose that an unusual, rapidly inactivating potassium conductance confers exceptional noise robustness on the system. We further investigate the possibility that a similar mechanism in other systems permits switching between plateau and spiking behavior under noisy conditions.

## Significance

We present evidence from computational modeling that an unusual K^+^ conductance endows the pharyngeal feeding organ of the nematode *Caenorhabditis elegans* with critical robustness against environmental noise that could otherwise disrupt its feeding strategy. This unusual K^+^ conductance inactivates very rapidly, which allows the generation of a sustained depolarized plateau until an inhibitory input hyperpolarizes the muscle sufficiently to remove the inactivation, allowing the K^+^ conductance to fully repolarize the muscle.

All animals need to eat, and worms are no exception. The nematode *Caenorhabditis elegans* feeds by a mechanism known as pharyngeal pumping (1). The pharynx is a large neuromuscular organ in the animal’s head that takes the form of a compartmented muscular tube of 20 muscle cells, innervated by its own nervous system of about the same number of neurons, together with additional nonneuronal cell types (2–5). This tube is subdivided into three segments: corpus, isthmus, and the terminal bulb (3). When the animal is feeding, the corpus opens and closes rhythmically, “pumping” water and bacterial food in, and then expelling only the water back out (1). Every several pumps, the isthmus moves peristaltically, driving bacteria posteriorly into the terminal bulb, and subsequently into the intestine. While this may appear straightforward, the pharyngeal system operates under severe biophysical constraints, and *C. elegans* has evolved ingenious solutions in response.

Perhaps most notably, the pharynx must solve a problem of timing. Because of their small size, and the even smaller size of their prey, *C. elegans* feeds in a low Reynolds number regime, where inertial forces are overridden by friction (6). Consequently, a symmetric pumping motion cannot achieve net transport of food through the pharynx and ultimately to the animal’s intestine. If the opening and closing motions are identical, food will only move back and forth, without proceeding posteriorly toward the isthmus. Failure to solve this problem of coordination is known to cause so-called hydrodynamic starvation in larval fish, resulting in the early death of the vast majority of live births (7). In *C. elegans* the solution to this physical impasse is that pharyngeal pumping is not fully symmetric (6). Although the muscles of the corpus open near-simultaneously, closing initiates from the anterior corpus, allowing for bacteria to become trapped and subsequently pushed back to the isthmus, where peristalsis can take over.

Thus to function properly, the pharynx must preserve certain relative timing, or phase, relationships. In particular, it is crucial that the main corpus not relax and close prematurely, before its anterior aspect does so independently. This conclusion is supported by previous work in which feeding was disrupted by laser killing the I5 interneuron, resulting in shortened pump duration (8, 9). This “slippery” pharynx opens and closes symmetrically and is unable to move bacteria posteriorly (10). More generally, it is known that a variety of drugs targeting neuromuscular signaling in the pharynx can be fatal to nematodes (11). However, even in a functioning pharyngeal nervous system, the biophysics of the excitable cells (neurons and muscle cells) involved present serious challenges for maintaining the phase relations necessary for the correct timing of pharyngeal pumping.

## Plateau Potentials and Environmental Noise

The main electrical event in the production of the pharynx’s pumping action is a myogenic plateau potential (2). Plateau potentials are long-lasting depolarizations from the cell’s resting membrane potential that outlast their precipitating impulse. They are found in a wide range of excitable cells, and perform diverse functions ranging from slow motor control (12–14) to the integration of stimuli distributed in time (15, 16). Plateaus are also implicated in certain pathological conditions, including chronic pain in spinal cord injury patients, further implicating this form of excitable dynamics as an important focus of study (17–19).

**Fig. 1.**
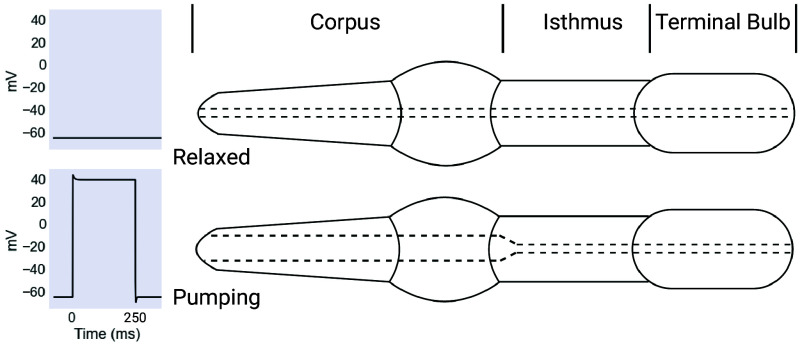
Pharyngeal pumping is driven by plateau potentials. The pharynx is a neuromuscular feeding organ composed of three principal segments: the corpus, the isthmus, and the terminal bulb. The pumping action of the pharynx is driven by myogenic plateau potentials. Plateaus correspond to a muscle contraction and an open corpus, as seen in the *Bottom* panel, while a resting membrane potential corresponds to a relaxed, closed corpus, depicted in the *Top* panel. Created with BioRender.com.

In *C. elegans*, a short EPSP arising from the paired MC motor neurons can depolarize the muscle cells of the pharyngeal corpus to nearly +40 mV for hundreds of milliseconds, driving the movement of ${\mathrm{Ca}}^{2+}$ ions needed for the muscles’ contraction (2). This coupling between plateau potentials and pharyngeal pumping is visualized in Fig. 1.

The worm’s problem of phase maintenance, then, reduces to a problem of ensuring that these plateaus are not prematurely terminated. This may present a significant challenge, because there is both theoretical and experimental evidence that the worm must accomplish this reliable timing under noisy conditions.

Under normal circumstances in a healthy wild type worm, plateau initiation and termination are timed by the paired MC excitatory cholinergic and M3 inhibitory glutamatergic motor neurons respectively (20). However, the biophysics of *C. elegans* neurons themselves should be expected to render these control signals extremely noisy. The small size of *C. elegans* neurons results in a very high input resistance, meaning that small current fluctuations caused by the random opening of ion channels can produce relatively large deflections in membrane potential, driving stochastic release of neurotransmitter that can then bind receptors on pharyngeal muscle (21).

There is significant theoretical evidence to support the claim that in cells and subcellular components with thousands of ion channels, such noise can have a substantial impact on membrane potential (22). In addition, the pharyngeal muscle also exhibits extremely high input impedance, meaning that its membrane potential is also highly susceptible to small, stochastic variations in the opening and closing of ionotropic receptor molecules (23). Given that a plateau of +40 mV would position the pharyngeal muscles well above the reversal potential of most mixed cation channels, the resulting noise would be hyperpolarizing, tending to bias plateaus toward early termination. These conclusions are also supported by recordings of electropharyngeogram (EPG) data, which often show hyperpolarizing deflections during the plateau phase of the pharyngeal pump (2).

## An Unusual Mechanism of Plateau Termination

Plateau potentials are a ubiquitous feature of nervous systems across the animal kingdom. However, despite this ubiquity and the rich diversity of functions they subserve, the fundamental elements of a plateau are quite consistent. To build a plateau potential, a cell needs a persistent inward current coupled with a delayed outward current (24). This delayed outward current is assumed or known to be a delayed rectifier or ${\mathrm{Ca}}^{2+}$-dependent potassium channel (14, 25). In other words, the outward current that ultimately repolarizes the cell begins integrating from the beginning of the plateau, building until the point where it overwhelms the persistent inward current resulting in plateau termination.

However, the mechanisms that operate to terminate the plateau in *C. elegans* appear to be different. The repolarizing $K^{+}$ conductance EXP-2 is a delayed rectifier that, in heterologous expression systems, exhibits ultrafast inactivation and slower deinactivation (23). However, in its native expression environment, this asymmetry between inactivation and deinactivation disappears, and the channel both inactivates and deinactivates very rapidly, with timescales close to 1 ms at relatively depolarized membrane potentials (23). The result is that the channel inactivates almost immediately upon depolarization and remains in a primed state, with the activation variable saturated, but the channel unable to pass current, until sufficient hyperpolarization relieves the inactivation, causing the channel to hyperpolarize the cell.

Below we investigate the functional consequences of these unusual channel kinetics for *C. elegans* pharyngeal muscle and show that they provide the pharynx with a crucial robustness against environmental noise.

## Materials and Methods

### The Model.

**Fig. 2.**
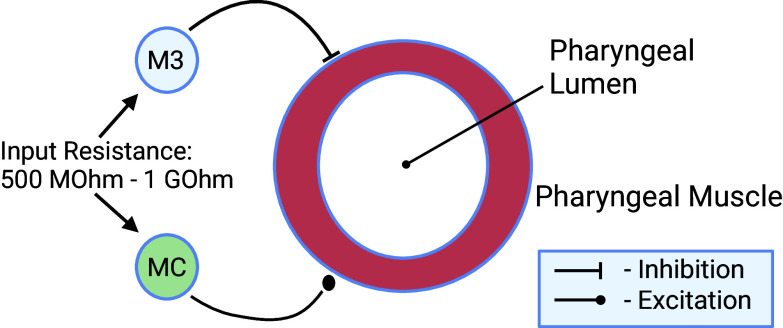
Model of the *C. elegans* pharynx. The model includes the motor neurons MC and M3 as respectively excitatory and inhibitory inputs to an electrically uniform pharyngeal muscle compartment. The noisy character of presynaptic signaling included in the model is understood to stem in part from the small size and high input resistance of these neurons. Created with BioRender.com.

The muscles of the *C. elegans* pharynx are innervated by 20 neurons that together form a nearly independent pharyngeal nervous system. However, laser killing experiments have demonstrated that not all of these are necessary for normal pumping. Based on these results, we implemented a reduced computational model of the pharynx (26), in which the MC and M3 motor neurons alone innervate the muscle cells of the pharyngeal corpus, which is represented as a single, electrically uniform compartment. This model is similar to the minimal qualitative model presented in ref. 23 and is visualized in Fig. 2.

This model was quantitatively implemented as a conductance-based model in the Hodgkin–Huxley formalism. The ultrafast inactivating potassium conductance that is the major focus of our interest was modeled as a simple potassium channel with instantaneous inactivation dynamics, given by

$$\begin{array}{c} I_{k}=-g_{k}wh_{\infty }\left(V-V_{k}\right), \end{array}$$

where $g_{k}$ is set to 9.0 pS, $V_{k}=-70.0\;\mathrm{mV}$, w is given as the solution to the ordinary differential equation

$$\begin{array}{c} \frac{\mathrm{dw}}{\mathrm{dt}}=0.04\frac{\left(w_{\infty }-w\right)}{\tau_{w}}, \end{array}$$

with $w_{\infty }$ and $\tau_{w}$ given by

$$\begin{array}{c} w_{\infty }=0.5\left(1+tanh\left(\left(V-12\right)/\left(17.4\right)\right)\right), \end{array}$$

and

$$\begin{array}{c} \tau_{w}=\frac{1}{cosh((V-12)/(34.8))}, \end{array}$$

and $h_{\infty }$ is our instantaneous inactivation modeled by

$$\begin{array}{c} 0.5(1-tanh\left(V+V_{\frac{1}{2}}/3\right)). \end{array}$$

In this expression, $V_{\frac{1}{2}}$ is the half-inactivation voltage, and was varied throughout in order to explore its impact on the dynamics of plateau generation and termination.

Experimental evidence suggests that the pharyngeal muscle expresses a T-type calcium channel gene *cca-1* as well as an L-type calcium channel gene *egl-19* (23, 27). In the absence of appropriate electrophysiological data for fitting a quantitative model of these specific channels in pharyngeal muscle, we modeled them based on generic prescriptions for these channel types found in ref. 28.

In addition to the ultrafast inactivating channel, *cca-1*, and *egl-19*, the model also includes a leak, excitatory, and inhibitory synaptic currents, all of which amounted to the following current balance equation.

$$\begin{array}{cc} C\frac{\mathrm{dV}}{\mathrm{dt}} & =-g_{L}\left(V-V_{L}\right)\;-g_{Ca_{T}}m^{2}h_{T}\left(V-V_{\mathrm{Ca}}\right)\\ & \;-g_{k}wh_{\infty }\left(V-V_{k}\right)-g_{Ca_{L}}h_{L}m_{L}^{2}\left(V-V_{\mathrm{Ca}}\right)\\ & \;-g_{\mathrm{synE}}\left(V-V_{\mathrm{syn}}\right)\;-g_{\mathrm{synI}}\left(V-V_{\mathrm{Cl}}\right)), \end{array}$$

where $V_{L}$ = −65.0 mV, $V_{\mathrm{Ca}}$ = 100.0 mV, $V_{\mathrm{synE}}$ = 0.0 mV, $V_{\mathrm{synI}}$ = −65.0 mV, $g_{L}$ = 2.0 nS, and $g_{Ca_{T}}$ = 3.0 nS, $g_{Ca_{L}}$ = 3.7 nS.

The maximal conductance for the two synaptic currents, $g_{\mathrm{synE}}$ and $g_{\mathrm{synI}}$ respectively, was set by assigning a maximal value at the time of each spike ($g_{\mathrm{synE}}=3.0$ nS and $g_{\mathrm{synI}}=2.5$ nS), which then decayed according to the differential equation

$$\begin{array}{c} \frac{\mathrm{dg}}{\mathrm{dt}}=-g/\tau , \end{array}$$

where $\tau_{E}=1.0\;\mathrm{ms}$ and $\tau_{I}=2.0\;\mathrm{ms}$.

The remaining gating variables are given as the solution to the differential equation of the form

$$\begin{array}{c} \frac{\mathrm{df}}{\mathrm{dt}}=\left(f_{\infty }-f\right)/\tau_{f}, \end{array}$$

where

$$\begin{array}{cc} m_{T_{\infty }} & =\frac{1}{2}\left(1+tanh\left(\left(V+45\right)/6\right)\right)\\ \tau_{m_{T}} & =\frac{1}{0.5cosh(V-14.5)/34.8}\\ h_{T_{\infty }} & =\frac{1}{2}\left(1-tanh\left(\left(V+40\right)/3\right)\right)\\ \tau_{h_{T}} & =\frac{65.0}{\left(1.0+e^{(V+40.0)/7.0}\right)}\\ m_{L_{\infty }} & =\frac{1.0}{1.0+e^{-(V+20.0)/6.5}}\\ \tau_{m_{L}} & =\frac{1.0}{1.0+e^{(V+27.0)/10.0}}\\ h_{L_{\infty }} & =200.0+\frac{100.0}{1.0+e^{(V+40.0)/7.0}}\\ \tau_{h_{L}} & =\frac{1.0}{1.0+e^{(V-135.0)/55.0}}. \end{array}$$

**Fig. 3.**
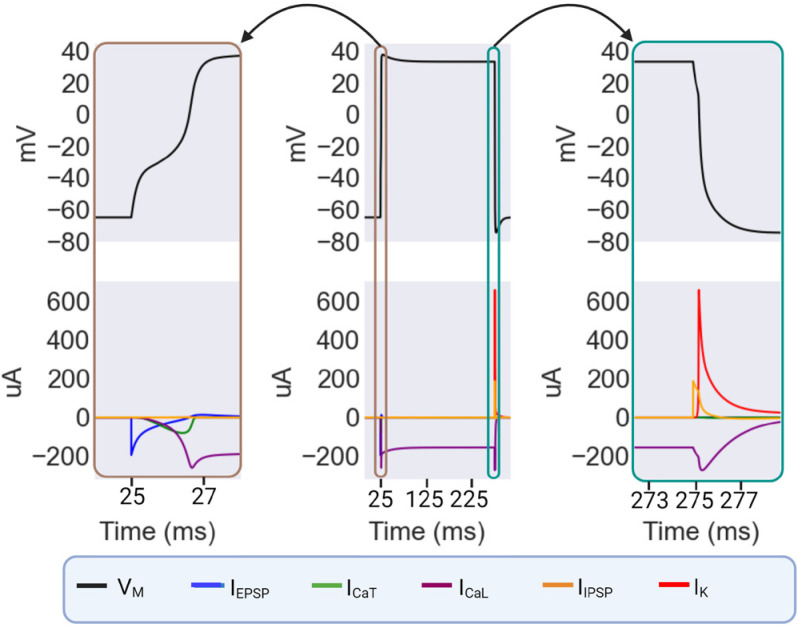
The ionic basis of plateaus in the model. The *Right* and *Left* panels are blow-ups of the indicated sections of the *Central* panel. In the *Leftmost* panel, the initiation of the plateau begins with an EPSP resulting from MC cell input. This drives the activation of a T-type calcium channel, which in turn drives the activation of an L-type calcium channel which is the basis of the long-lasting plateau itself. In the *Rightmost* panel, an IPSP resulting from input from the M3 cell hyperpolarizes the muscle enough to remove inactivation from the EXP-2 $K^{+}$ channel, which subsequently causes the repolarization of the cell. Created with BioRender.com.

This model generates plateau potentials as summarized in Fig. 3. The plateau is initiated by an EPSP, which activates the T-type calcium conductance based on the CCA-1 channel and the L-type conductance based on the EGL-19 channel in sequence. The L-type conductance is responsible for the sustained depolarized phased of the plateau. Finally, the end of the plateau is timed by the arrival of an IPSP. Until the rapid hyperpolarization by the IPSP, the EXP-2 $K^{+}$ conductance is in a primed inactivated state, with the inactivation variable h locked at zero and the activation variable w saturated at one. Due to the ultrafast kinetics of the inactivation variable, the short IPSP is enough to deinactivate the conductance, driving the emergence of a hyperpolarizing current and repolarizing the cell.

### Long Timescale Models.

In order to explore the role of the timescale of inactivation, we also relaxed the assumption of instantaneous inactivation for the EXP-2 $K^{+}$ conductance. In these models, the form of $h_{\infty }$ given above was preserved, but the current due to EXP-2 was calculated as

$$\begin{array}{c} I_{k}=-g_{k}wh\left(V-V_{k}\right), \end{array}$$

with h given by the solution to the ordinary differential equation

$$\begin{array}{c} \frac{\mathrm{dh}}{\mathrm{dt}}=\frac{\left(h_{\infty }-h\right)}{\tau_{h}}. \end{array}$$

The values chosen for $\tau_{h}$ were 1, 5, 10, and 20 ms, which spanned a range of interesting behaviors.

### Simulations.

Individual simulations based on the model were carried out in the python programming language, using an LSODA solver to solve the system of differential equations. Three versions of the model, which differed in the half-inactivation voltage of the ultrafast inactivating potassium current ($V_{1/2}=10,15,20\;\mathrm{mV}$), were run for 1,000 s of model time each. These simulations were each performed in low and high noise conditions.

### Noise.

For the investigation of noise robustness, noise was assumed to come from fluctuations in the membrane potential of presynaptic neurons. Because of the small size of *C. elegans* neurons, and their resulting high input resistance and small number of ion channels, they are especially susceptible to noisy fluctuations in membrane potential (21). This is assumed to result in the release of small amounts of neurotransmitter into the synaptic cleft.

Although there is evidence for the existence of both excitatory and inhibitory noise in this system, we chose to model only inhibitory noise. We made this choice for two reasons. First, excitatory current noise based on the opening of channels becomes inhibitory when the membrane potential of the cell rises above the reversal potential of the relevant channel. The excitatory input was modeled as a mixed cation conductance with a reversal potential of 0 mV. Thus the resulting noise would be inhibitory for the duration of the plateau. Second, in the interest of conceptual clarity, because the focus of this study is the role of an unusual conductance in shaping plateau termination, we made the decision to focus on the noise polarity relevant to the plateau.

To model this transmission noise computationally, we represented the random release of neurotransmitter as shot noise, using a Poisson process to generate a series of arrival times and numbers of open channels, which were then multiplied by a constant value (0.15 nS) representing the contribution of the noise event to presynaptic conductance, which was then introduced as fluctuations in the synaptic conductance in the current balance equation during simulation at the arrival times sampled from the Poisson distribution using the Poisson function in Python’s NumPy package. Because noise events were Poisson distributed, the probability of seeing k noise events in a given time interval is distributed as follows.

$$\begin{array}{c} P\left(x=k\right)=\frac{\lambda^{k}e^{-\lambda }}{k!}, \end{array}$$

where λ functions as a characteristic rate of occurrence. In our model, we used this parameter to set the noise intensity, with λ=0.01 corresponding to low noise, and λ=0.05 corresponding to high noise.

### MC and M3.

The dynamics of the presynaptic cells was computationally represented as a hard coded list of event times for EPSPs and IPSPs in the explicitly modeled pharyngeal muscle. Absent the influence of noise, these lists were chosen to produce regular series of plateaus with a given length and interspike interval. In what follows plateau length was chosen to be 250 ms, with an ISI of 250 ms, which is broadly similar to the rate of pharyngeal pumping during active feeding.

## Results

### Characterization of the Model Under Noiseless Conditions.

To characterize the behavior of the model without noise, we varied the maximal conductances of the $I_{K}$ and $I_{Ca_{L}}$ currents between 0 and 10 nS and 0 and 7.5 nS, respectively. Because these two currents provide the sustained inward and delayed outward currents that are the core elements of a plateau, covarying them provides a broad view of the noiseless model’s behavior.

**Fig. 4.**
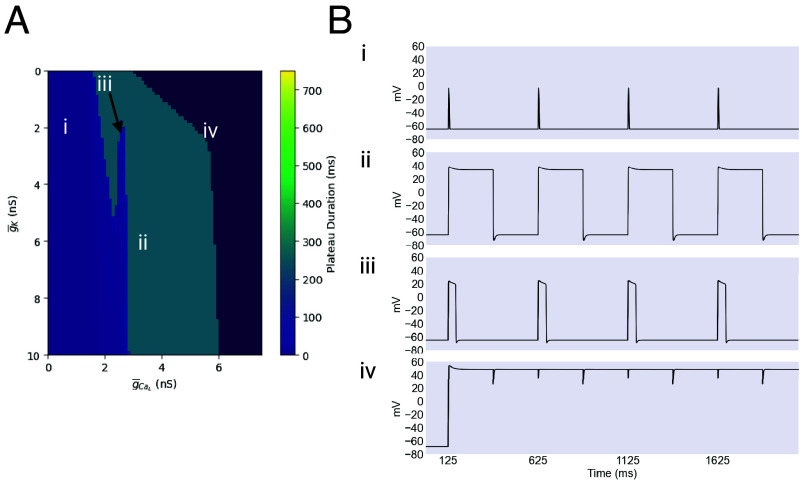
The ${\overset{¯}{g}}_{K}/{\overset{¯}{g}}_{Ca_{L}}$ parameter space. (*A*) The mean plateau duration is plotted for a range of values of the maximal conductances of $I_{K}$ and $I_{Ca_{L}}$. The leftmost portion of the plot, shown in light purple, corresponds to a continuum of behaviors ranging from low-amplitude spikes to low-amplitude, short-duration plateaus. The rightmost portion, shown in dark purple, represents combinations of conductances that result in a complete failure of rhythmicity, producing unending plateaus. For scaling reasons, this region is colored without reference to the colorbar. Finally, the turquoise central region represents the region of physiological function. (*B*) Four exemplar traces of the behavior found in the regions described above are shown. Created with BioRender.com.

The results of this parameter sweep are shown in Fig. 4. The broad turquoise band down the center of Fig. 4*A* represents the zone of physiological behavior, where plateaus are accurately timed by excitatory and inhibitory neuronal input. The rightmost portion of the space, shown in dark purple, corresponds to combinations of the maximal conductances that fail to produce rhythmic behavior at all, instead generating a single unending plateau, while the leftmost portion, shown in lighter purple, corresponds to a continuum of behaviors from low-amplitude spiking to short, low-amplitude plateaus.

An interesting feature of this figure is the relative insensitivity of the model’s behavior to the maximal conductance of $I_{K}$, with almost the full range of values able to produce functional behavior for a reasonably wide range of values of the maximal conductance of $I_{Ca_{L}}$ (Fig. 4*B*). This may have the consequence of rendering the system very flexible. The maximal conductances of ion channels can be extensively modulated by influences including temperature, pH, and neuromodulation, so the ability to produce functional plateaus over a wide portion of conductance space is likely valuable (29–31).

### Noise and the Role of the *V*_1/2_ of Inactivation.

**Fig. 5.**
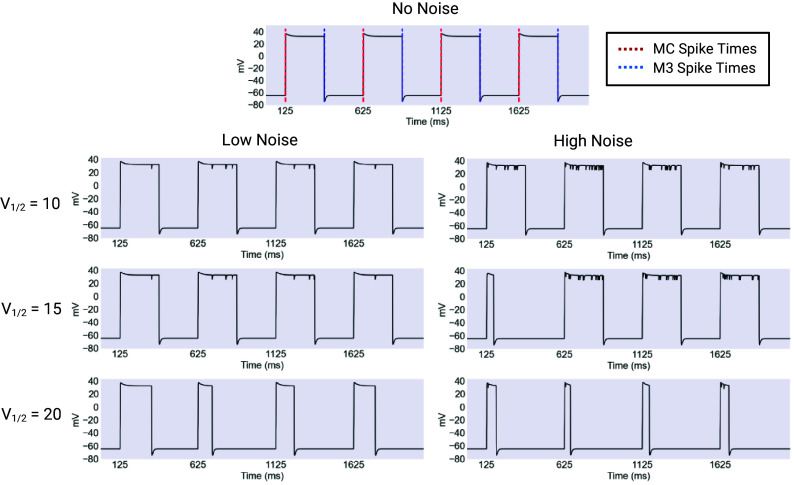
Example outputs of the ultrafast model. Output of the model in clean and noisy conditions for varying values of the $V_{1/2}$ of inactivation for the EXP-2 $K^{+}$ conductance. The model pharyngeal muscle was timed by EPSPs and IPSPs from the modeled MC and M3 cells respectively to produce 250 ms plateaus, close to the plateau length observed during feeding. The model produces identical output for all values of $V_{1/2}$ under noiseless conditions, represented in the *Top* panel. When noise is applied to the model, the versions with more hyperpolarized value of $V_{1/2}$ are more robust, while plateaus in models with relatively depolarized values are terminated early. This divergence between versions of the model becomes more pronounced under more intense noise. Created with BioRender.com.

Next, we simulated three versions of the model, which differed only in the half-inactivation voltage of the ultrafast inactivating potassium current ($V_{1/2}=10,15,20\;\mathrm{mV}$). The simulations were run for 1,000 s of model time each. These simulations were each performed in low and high noise conditions, corresponding to Poisson λ parameters of 0.01 and 0.05, respectively. The results, shown in Fig. 5, show clearly the impact of shifting the repolarizing current’s half-inactivation voltage. In the no noise condition, the models produce identical results. However, at low noise levels, the three models begin to diverge in performance. The $V_{1/2}=10\;\mathrm{mV}$ and $V_{1/2}=15\;\mathrm{mV}$ models remained able to robustly produce functional plateaus with the desired duration, while plateaus in the $V_{1/2}=20\;\mathrm{mV}$ model were very noticeably suppressed, with the average duration falling below 125 ms, or half the desired duration. As noise is increased, the divergence became more pronounced. The $V_{1/2}=10\;\mathrm{mV}$ remained largely immune to noise, with only a small number of premature terminations, resulting in a significant, but very slight, reduction in the mean plateau length, as shown in Fig. 5. Plateaus in the $V_{1/2}=15\;\mathrm{mV}$ model were more noticeably affected and terminated earlier on average than the intended 250 ms. Meanwhile plateaus produced by the $V_{1/2}=20\;\mathrm{mV}$ model were even more suppressed than in the low noise case, with the average duration falling to below 50 ms.

For both the low (λ = 0.01) and high (λ = 0.05) noise conditions, the effect size of the noise treatment on each model ($V_{1/2}$ = 10, 15, and 20 mV) was assessed using the two-tailed Mann–Whitney U test to derive a z-score relative to the noiseless case, which was used to calculate an effect size as $Effect=\frac{\left|z\right|}{\sqrt{N}}$, where N was the number of plateaus. In the low noise condition, the effects on the $V_{1/2}$ = 10 mV and 15 mV models were minuscule, while the effect on the $V_{1/2}$ = 20 mV model was pronounced. In the high noise case, the effect size for the $V_{1/2}$ = 10 mV remained negligible, while the effect size for the $V_{1/2}$ = 15 and 20 mV models was pronounced.

**Fig. 6.**
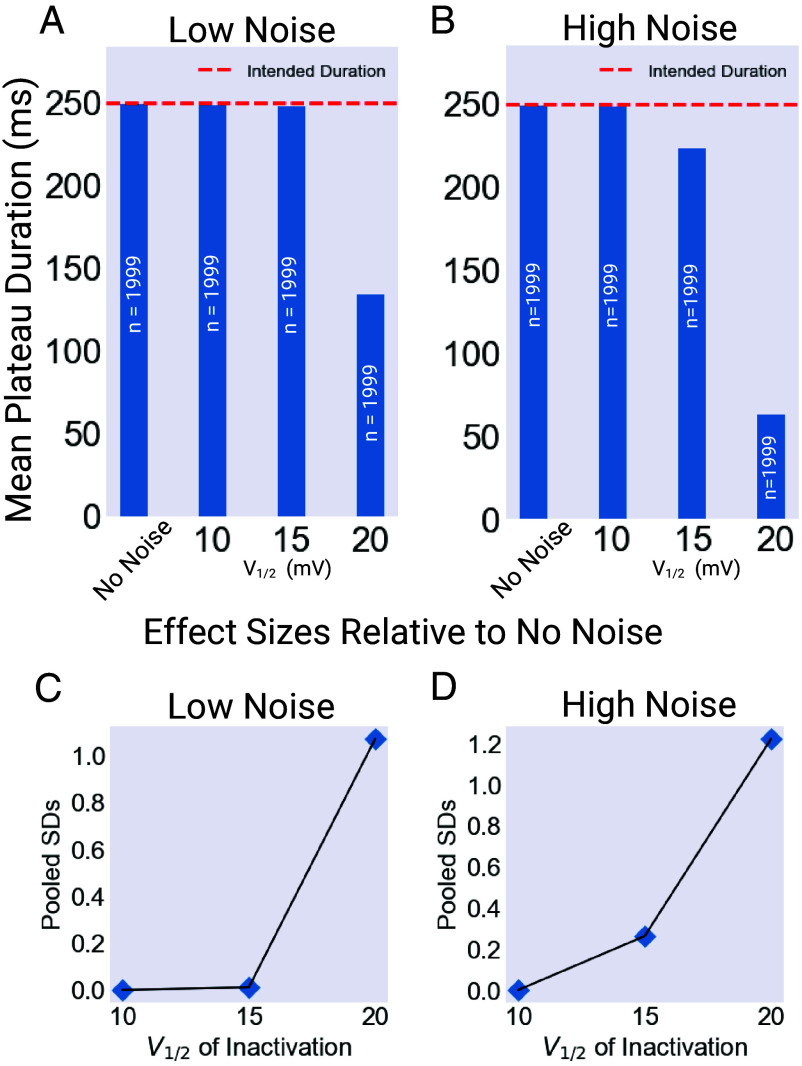
Summary of model outputs. Models with $V_{1/2}$ = 10, 15, 20 mV were run for 1,000 s of model time, and the length of the resulting plateaus averaged for low (λ=0.01) and high (λ=0.05) noise intensities. (*A*) Mean plateau duration for each model under low noise conditions. The “No Noise” Value was identical for all models. (*B*) Mean plateau duration for each model under high noise conditions. Again, the No Noise Value was identical for all models. (*C*) Effect size for each of the three models under low noise conditions. These values were calculated using the z-score of a two-tailed Mann–Whitney U test comparing the low noise and no noise results for each model. The effect size was calculated as $Effect=\frac{\left|z\right|}{\sqrt{N}}$. (*D*) Effect size for each of the three models under high noise conditions. These values were calculated using the z-score of a two-tailed Mann–Whitney U test comparing the high noise and no noise results for each model. The effect size was calculated as $Effect=\frac{\left|z\right|}{\sqrt{N}}$. Created with BioRender.com.

These results reveal a simple pattern. The three model versions are identical under noiseless conditions, but diverge in behavior as noise increases, as show in Fig. 6. Even under low noise levels, a change in the half-inactivation voltage of the repolarizing current can cause a collapse in plateau duration, driving a transition from regular plateaus to a more erratic regime characterized by variable-duration spikes. This effect is even more pronounced under high noise conditions. In vivo, this change could possibly be caused by neuromodulatory input (14, 32–34).

**Fig. 7.**
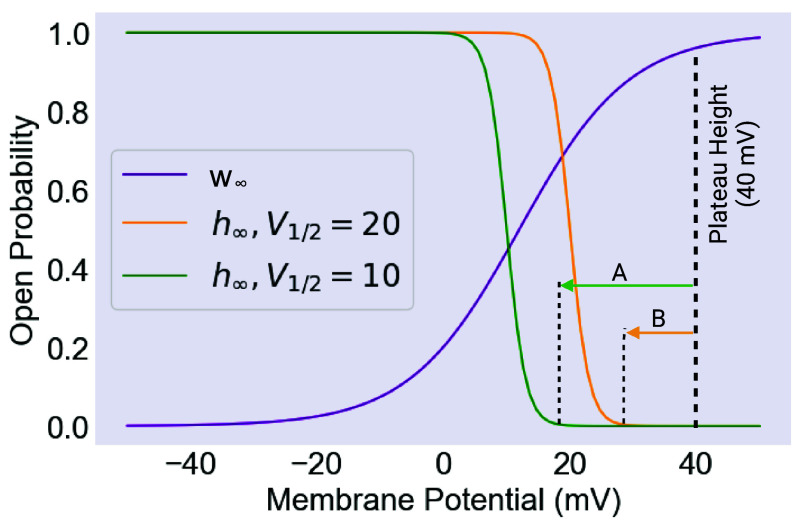
The mechanism of noise robustness. The activation ($w_{\infty }$) and inactivation ($h_{\infty }$) curves for EXP-2. The size of the EXP-2 conductance scales with the product of $w_{\infty }$ and $h_{\infty }$, meaning that the bigger the nonzero overlap of the two curves, the larger the hyperpolarizing current being passed. Consequently, moving the $h_{\infty }$ curve horizontally influences the excitability of the EXP-2 conductance. (*A*) If the plateau is holding at 40 mV, and $h_{\infty }$ is given by the turquoise curve, then a larger initial hyperpolarization is required to drive the cell to a potential where $h_{\infty }$ is nonzero, allowing EXP-2 to conduct and hyperpolarize the cell. (*B*) If the plateau is holding at 40 mV, and $h_{\infty }$ is given by the yellow curve, then a smaller initial hyperpolarization is required to drive the cell to a potential where $h_{\infty }$ is nonzero, allowing EXP-2 to conduct and hyperpolarize the cell. In case *A*, a larger noise event would be required to activate EXP-2, and in case *B* a smaller one. Consequently, the more left shifted curve, with the more hyperpolarized value of $V_{1/2}$, is more robust to noise. Created with BioRender.com.

These results, in particular the importance of the $V_{1/2}$ value, suggest an interpretation that can be understood by examining the plot of the EXP-2 activation and inactivation curves in Fig. 7. As seen in the figure, $V_{1/2}$ value controls the horizontal position of the inactivation $h_{\infty }$ curve relative to the activation $m_{\infty }$ curve. When the $V_{1/2}$ value is relatively depolarized, the $h_{\infty }$ curve is shifted rightward, with the consequence that nonzero regions of the $h_{\infty }$ curve are closer to the level of the plateau potentials. For the plateau to repolarize, inhibitory input from the M3 cells must drive the cell to a potential where $h_{\infty }$ is nonzero, deinactivating the EXP-2 current and driving the cell back down to resting potential. This can be accomplished either by an action potential or by noise events. The key point is that this task is made easier or harder by changes in the value of $V_{1/2}$.

### Timescales of Inactivation.

A natural question is whether the ultrafast inactivation in our model is necessary for proper function in general, and for noise robustness in particular. In order to answer this question we introduced a model which differed from the ultrafast model only in having a constant timescale for inactivation, which was set at 1, 5, 10, and 20 ms.

**Fig. 8.**
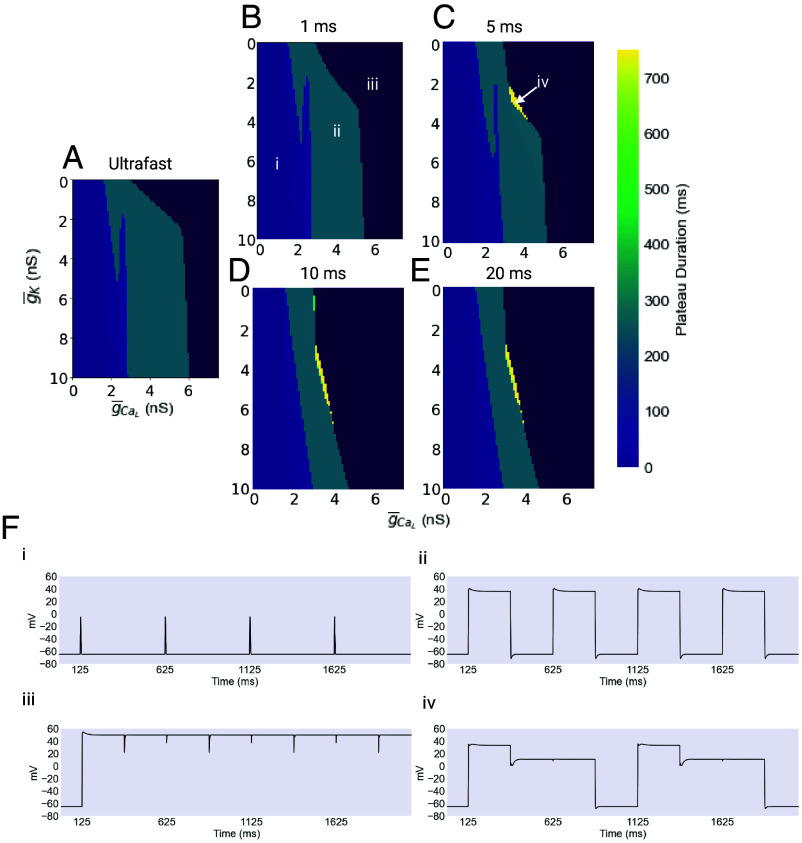
The ${\overset{¯}{g}}_{K}/{\overset{¯}{g}}_{Ca_{L}}$ parameter space in longer timescale models. (*A*–*E*) The mean plateau duration is plotted for a range of values of the maximal conductances of $I_{K}$ and $I_{Ca_{L}}$. The *Leftmost* portion of the plot, shown in light purple, corresponds to a continuum of behaviors ranging from low-amplitude spikes to low-amplitude, short-duration plateaus. The *Rightmost* portion, shown in dark purple, represents combinations of conductances that result in a complete failure of rhythmicity, producing unending plateaus. For scaling reasons, this region is colored without reference to the colorbar. The turquoise central regions represent the region of physiological function. Finally, yellow patches represent regions of long duration, two-level plateaus. (*F*) Four exemplar traces of the behavior found in the regions described above are shown. Created with BioRender.com.

We found that the introduction of a slower timescale had significant consequences for the production and maintenance of plateaus. As with the ultrafast model, we first explored the behavior of these models for different values of the maximal conductances of $I_{K}$ and $I_{Ca_{L}}$, ranging from 0 to 10 nS and 0 to 7.5 nS, respectively. These simulations were all performed with a $V_{1/2}$ of inactivation of 15 mV. The results are summarized in Fig. 8, and show important differences in the behavior of these models relative to the ultrafast model.

As before in the ultrafast case, the dark purple dominating the far right of each panel represents combinations of maximal conductances where the resulting plateau does not terminate, and rhythmicity is entirely lost. The central turquoise stripe represents the zone of physiological function, and the light purple region of the left represents low-amplitude spiking.

An unusual possibility that appears only in the longer timescale models is the two level plateau. Seen in the yellow regions of the plots above, these plateaus begin close to 40 mV and persist there for several hundred milliseconds before falling to a second plateau level near 0 mV and remaining there for several hundred more milliseconds, before finally returning to resting membrane potential. A possible consequence of this two-level plateau is an initial, partial closure of the pharynx, resulting in expulsion of some food. As a result, this electrical behavior may reduce the viability of the animal by reducing the efficiency of feeding.

There are several interesting things about these results. In the first place, the 1 ms model is very similar, although not identical, to the ultrafast model. In each case, there is a broad range of functional values. This range, however, narrows considerably as the timescale of inactivation increases, especially with respect to the value of ${\overset{¯}{g}}_{Ca_{L}}$. For the slower models, the band of functional combinations also takes on a slant, which reduces the range of values of ${\overset{¯}{g}}_{K}$ that can work for a given value of ${\overset{¯}{g}}_{Ca_{L}}$. As mentioned above, this may have more limited range of functionality and may have consequences for modulation.

### Noise Robustness of the Long Timescale Models.

**Fig. 9.**
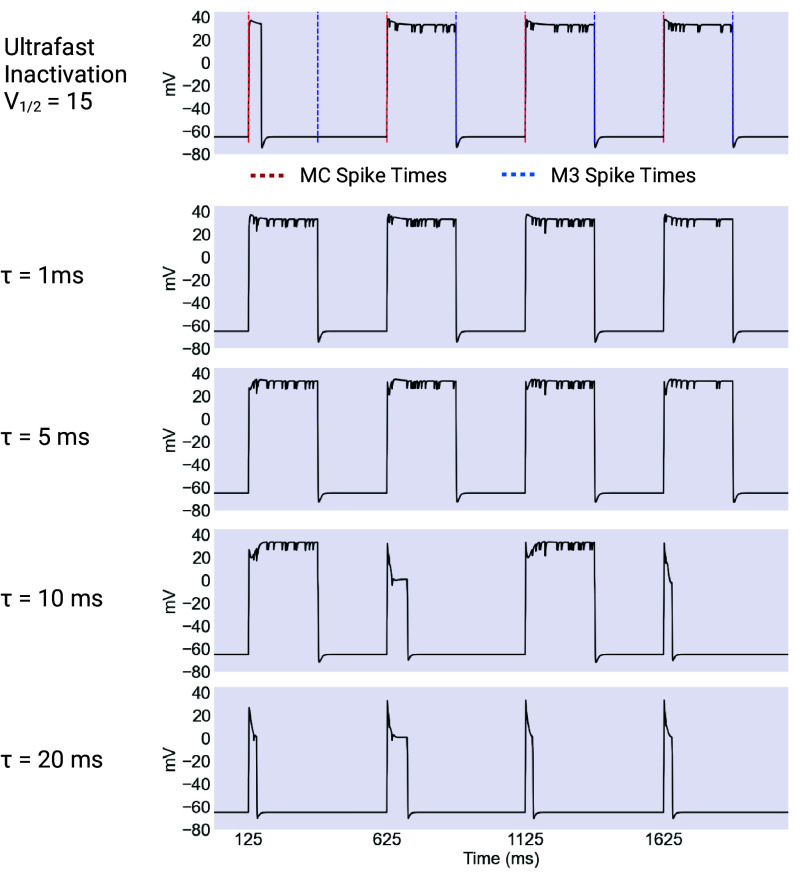
Example outputs of long timescale models. The ultrafast inactivation model compared to models with constant values of $\tau_{h}$. The ultrafast inactivating model produces mostly untruncated plateaus of the desired length of 250 ms, timed by input from the excitatory MC and inhibitory M3 cells. Note that the first plateau in the ultrafast was truncated early while all plateaus in the 5 and 10 ms cases were untruncated. When $\tau_{h}$ is set to 1 ms, the resulting plateaus are essentially identical to the ultrafast case. However when the value of $\tau_{h}$ is increased to 5 ms, the plateaus develop a noticeable “wedge” hyperpolarization immediately after their initial depolarization. At $\tau_{h}$ = 10 ms, the resulting plateaus are mostly truncated very early, and in surviving plateaus, this wedge hyperpolarization is more pronounced. Created with BioRender.com.

**Fig. 10.**
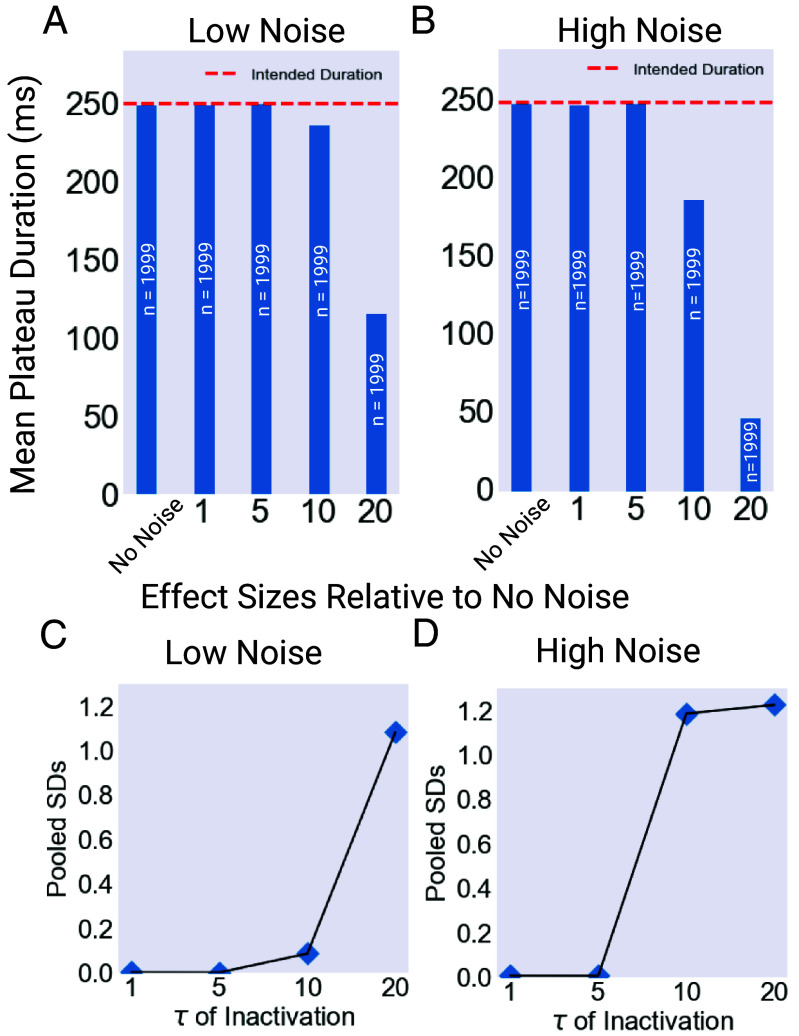
Summary of model outputs. Models with τ = 1, 5, 10, and 20 ms were run for 1,000 s of model time, and the length of the resulting plateaus averaged for low (λ=0.01) and high (λ=0.05) noise intensities. (*A*) Mean plateau duration for each model under low noise conditions. The No Noise Value was identical for all models. (*B*) Mean plateau duration for each model under high noise conditions. Again, the No Noise Value was identical for all models. (*C*) Effect size for each of the three models under low noise conditions. These values were calculated using the z-score of a two-tailed Mann–Whitney U test comparing the low noise and no noise results for each model. The effect size was calculated as $Effect=\frac{\left|z\right|}{\sqrt{N}}$. (*D*) Effect size for each of the three models under high noise conditions. These values were calculated using the z-score of a two-tailed Mann–Whitney U test comparing the high noise and no noise results for each model. The effect size was calculated as $Effect=\frac{\left|z\right|}{\sqrt{N}}$. Created with BioRender.com.

We next explored the behavior of the longer timescale models against a noisy background, using the same procedure as before. The longest timescales, 10 and 20 ms, resulted in plateaus that were much less robust to noise than in the ultrafast inactivating model. The shorter timescale models, on the other hand, were actually more robust to noise than the ultrafast model, with the 5 ms model being almost completely insensitive to noise in absolute terms. These results are summarized in Figs. 9 and 10.

As in the ultrafast models, the model was run for 1,000 s of model time for each time scale, and the effect size of the noise treatment was calculated for both the low (λ = 0.01) and high (λ = 0.05) noise conditions. In the low noise condition, the effect on the τ = 1 and 5 ms models were minuscule, while the effect on the τ = 10 ms model was marginal, and the effect on the τ = 20 ms model was pronounced. In the high noise case, the effect size for the τ = 1 and 5 ms models remained negligible, while the effect size for the τ = 10 and 20 ms models was pronounced.

**Fig. 11.**
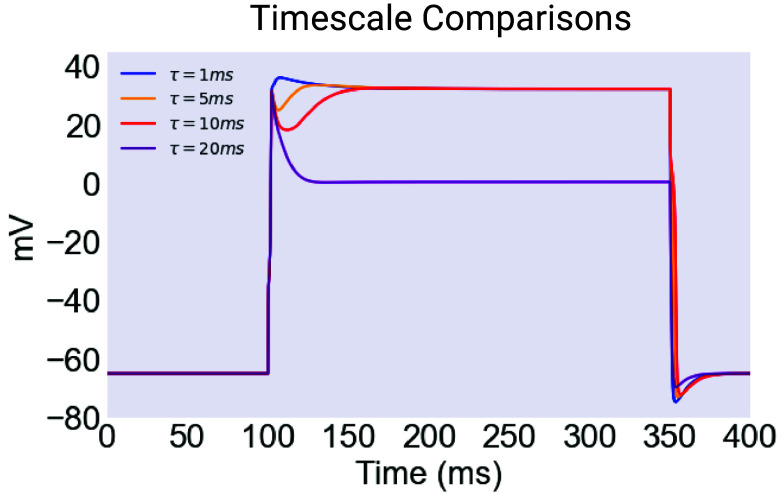
The structure of plateaus from longer timescale models. A single myogenic plateau as produced by four different models, each with a different value for $\tau_{h}$ of EXP-2. The shortest value of 1 ms produces plateaus essentially indistinguishable from the ultrafast model presented above, with a long relaxation from an initial peak, driven by EGL-19 inactivation. However as the timescale ($\tau_{h}$) is lengthened, a wedge hyperpolarization develops after an initial peak. This not only makes these plateaus more susceptible to noise events early in the course of the plateau. It may also impact the ability of the plateau to drive a consistent and appropriately timed contraction of the pharyngeal muscle. Created with BioRender.com.

It is instructive to study the shape of the plateaus generated by the long timescale models. In the 1 ms case, the plateaus are practically identical to those produced by the ultrafast model. This is expected, since in vivo 1 ms is practically instantaneous. However as the timescale lengthens, noticeable differences emerge. In particular, in the 5 ms model, we can see the emergence of a early wedge of hyperpolarization at the start of the plateau. These dynamics are summarized in Fig. 11.

The wedge grows larger in the 10 ms case and is likely responsible for the loss of robustness in this model, since it holds the cell relatively close to the threshold of deinactivation for EXP-2 for an extended period of time, making early termination much easier.

In the case of the 20 ms model, the wedge is deep enough that it forces the cell down to a lower-level plateau near 0 mV, where it is extremely sensitive to noise.

The excellent noise robustness of the 1 and 5 ms models is also interesting. It seems to represent a balance between the depth of the “hiccup” hyperpolarization, which is small in these two cases, and the ability of the longer timescale of inactivation (1 and 5 ms as opposed to instantaneous) to essentially filter out fast hyperpolarizing noise events.

This filtering depends on the relationship between the timescale of recovery from inactivation for EXP-2 and the characteristic length of noise events, which are given by $\tau_{h}$ and τ (the timescale of the inhibitory M3 synapse) respectively. When the timescale of recovery from inactivation is long relative to the length of a noise event, EXP-2 is not able to recover before the precipitating hyperpolarization ends, meaning that the impact of noise is reduced. This, combined with the fact that the initial wedge hyperpolarization is not deep, may explain the noise robustness of plateaus in these models.

**Fig. 12.**
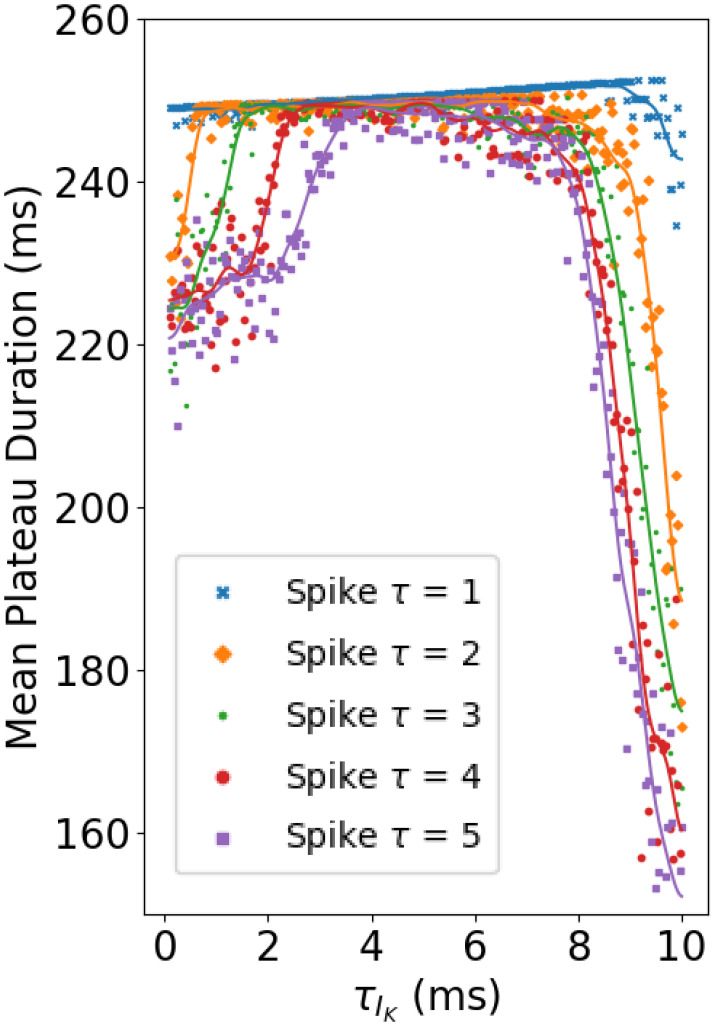
Interactions between synaptic and inactivation timescales. Faster timescale noise events are readily filtered out by cells with slower timescale $I_{K}$ inactivation. Each curve corresponds to the mean plateau duration over 100 seconds of model time for models with time constants of inactivation of $I_{K}$ ranging from 0.5 to 10 ms, for a given value of the synaptic time constant, which controls the timescale of inhibitory noise events. For faster noise events, models with slower values of $\tau_{I_{K}}$ effectively filter out noise, producing relatively robust plateaus. This effect is counterbalanced by the initial hiccup hyperpolarization produced by slower inactivation timescales, which at larger values of $\tau_{I_{K}}$ renders plateaus less robust. Created with BioRender.com.

To further establish this hypothesis, we ran the model for a range of values of $\tau_{h}$ of inactivation, while also varying the synaptic timescale τ. The results are shown in Fig. 12. In the figure, each curve corresponds to a particular time scale for noise events, ranging from 1 to 5 ms. For each value, the curve contains a region of robustness, where plateaus are protected against noise for a range of values of timescale of EXP-2 inactivation. This is the flat region of each curve, where plateaus are maintained at roughly the desired length of 250 ms for a range of values of $\tau_{h}$. As the timescale of noise events lengthens, this region shrinks from the left, reflecting the inability of faster models to adequately filter out longer noise events.

On the right side, robustness falls again, reflecting the increasing depth of the initial hiccup hyperpolarization in plateaus produced by longer inactivation timescale models.

## Discussion

### How the Worm Churns.

In this study, we explored the consequences of an unusual potassium conductance, EXP-2, for the duration and robustness of plateau potentials in a simple conductance-based model of the *C. elegans* pharynx. The ultrafast inactivating potassium conductance explored in this study represents a clever trick of evolution. By turning itself off from the start, this conductance allows the production of arbitrarily long plateaus. This feature of the model is supported by experimental evidence. In worms with *exp-2* loss of function mutations, plateaus can stretch on for several seconds (23, 35, 36). Similar plateau lengths have been observed in worms in which the glutamatergic inhibitory M3 motor neurons have been killed.

The intrinsic potential for long duration plateaus facilitated by EXP-2’s unusual kinetics places the timing of plateau termination at the discretion of presynaptic neurons, and thus gives the organism enormous and crucial flexibility and dynamic range in the frequency and phase behaviors of resulting rhythmic muscular actions. It has been previously demonstrated that failures of phase maintenance in the *C. elegans* pharynx can lead to serious deficits in feeding, resulting in starved worms (1).

In addition to the ability to generate plateaus of any desired length, this system proves to be highly resilient to modeled fluctuations in the synaptic inputs to the simulated muscle. The ultrafast inactivation of EXP-2 has the effect of placing the channel in a primed, but inactivated, state, in which it can sit indefinitely. At the same time, while the channel is in this state a sufficient hyperpolarization can readily drive it to rapidly repolarize the cell. The size of the required hyperpolarization is set by the value of the $V_{1/2}$ of inactivation, or in other words by the horizontal position of the inactivation curve. This effective hyperpolarization threshold for plateau termination has the effect of filtering out noisy hyperpolarizing inputs, while leaving the cell ready to respond to neural timing signals from the M3 cells. In the absence of experimentally determined values, we chose the range of $V_{1/2}$ in order to demonstrate a range of dynamics.

In addition to the value of the $V_{1/2}$ of inactivation, we found that this behavior also depends on the time constants of inactivation and deinactivation. Based on previous experimental work, we assumed here that inactivation and deinactivation were symmetric processes with a shared time constant. In our primary model, we assumed instantaneous inactivation and deinactivation. Relaxing this assumption by adding a nonzero time constant to these processes revealed an interesting and diverse range of dynamics. In the noiseless case, the longer timescale models revealed a limited functional range within their maximal conductance parameter space.

When noise was added to these models, we found a biphasic response to increased time constants. Noninstantaneous but still fast inactivation, with τ = 1 or 5 ms resulted in remarkable noise robustness, outperforming even the ultrafast model. Further exploration revealed that this resulted from the ability of more slowly responding models to act as low-pass filters, effectively screening out fast noise events. However, at longer time scales, in models with τ = 10 or 20 ms the robustness of plateaus against a noisy background was reduced, owing to an initial wedge of hyperpolarization at the start of each plateau. These values of τ were chosen to span a range of fast but not instantaneous dynamics, to provide a point of comparison for the ultrafast model.

This increased sensitivity resulted from wedge hyperpolarizations that resulted directly from the slower inactivation of EXP-2. When this current inactivates slowly, an overlap in nonzero values of the inactivation and activation gating variables develops, producing a current which transiently hyperpolarizes the cell, making it temporarily more susceptible to noise.

In addition to increased noise sensitivity, such wedge hyperpolarizations are likely dysfunctional in themselves. Given that the contraction of muscle correlates closely with membrane potential, the “stutter step” beginning to plateaus observed in models with relatively long time constants would likely have negative consequences on feeding and thus the viability of the worm.

### Comparisons to Other Systems.

EXP-2 has similar kinetics to several other channels known from mammalian systems, such as the human *ether-a-go-go* (hERG) conductance (37) which mediates the final repolarization of the cardiac action potential. It has previously been suggested that this characteristic is essential for precise and rapid repolarization of muscular actions (23), but our analysis suggests that this property may also contribute to reliability of muscle function in the face of random fluctuations.

More generally, the results summarized in Fig. 12 suggest that the mechanism explored in this report does not strictly require instantaneous inactivation. This mechanism also functions for more slowly inactivating systems, depending on the relationship between the timescale of inactivation and the frequency content of the noise to which the system is subjected. Furthermore, a version of this mechanism should still be operative wherever inactivation is fast relative to activation. Both of these facts have implications for the broader application of this model to understanding other systems.

### A Potential Mechanism for Gating Plateaus.

Even though this model was based on the *C. elegans* pharynx, its findings are potentially of much broader interest. Plateau potentials are involved in an enormous range of physiological processes. Electrical behavior similar to that found in *C. elegans* pharynx is also observed in dendrites during the formation of hippocampal place fields (38, 39), as well as in the crustacean ventilatory system, the thalamus, and mammalian spinal motor neurons (13, 40, 41).

Importantly, most of these systems do not express plateau potentials constitutively. Plateaus are gated by the cell and activated for particular purposes when circumstances demand it. This should not be surprising, since plateau potentials can correspond to a long-lasting increase or decrease in the baseline excitability of a cell, depending on its particular electrophysiology. In fact, persistent dysregulated plateau potentials have been linked to pathological conditions, including chronic pain and spasticity in victims of chronic spinal cord injury.

It seems possible that other systems may implement a noise-based mechanism like that described in this study for selectively gating the generation of plateaus. Noise is an abundant resource even in nervous systems with very different biophysics from *C. elegans*, especially in systems which experience balanced excitatory and inhibitory inputs. For instance, although mammalian cortical pyramidal neurons are much larger than the neurons found in *C. elegans*, these cells typically receive thousands of largely uncorrelated inputs (42, 43). Motor neurons in an ex vivo prep of the turtle spine similarly have been found to receive many uncorrelated inputs (33). Previous theoretical work has shown that such an arrangement has a tendency to promote the importance of fluctuations in inputs (42).

At the same time, plateaus are known to be actively suppressed in some systems. Although the details of this suppression remain obscure, in some settings, it is known to depend on neuromodulatory input. In particular, spasticity caused by errant plateau potentials in rats is known to be dependent on the loss of top–down neuromodulatory control from the brain stem resulting from spinal cord injury (18, 44).

Given these results, the model presented in this paper, in which a shift in the inactivation curve of a $K^{+}$ current acts as a gate on the production of plateau potentials, may be an appropriate model for neuromodulation-induced selective suppression and production of plateaus.

## Data Availability

Code has been deposited in GitHub (26).
